# Touchdown-singularity formation and criticality in the thin-film equation

**DOI:** 10.1098/rsta.2023.0375

**Published:** 2025-05-08

**Authors:** John King, Mark Bowen

**Affiliations:** ^1^ School of Mathematical Sciences, University of Nottingham, Nottingham, UK; ^2^ Waseda University Faculty of Science and Engineering, Shinjuku-ku, Tokyo, Japan

**Keywords:** self-similarity, singularity formation, film rupture, criticality

## Abstract

The thin-film equation (TFE), $h_{t}=-(h^{n}h_{xxx}{)}_{x}$, is significant physically in the description of surface-tension-driven flows of thin films of viscous liquids and has served an important role mathematically in elucidating the properties and challenges of high-order degenerate parabolic equations. Long-standing open questions nevertheless remain, of which perhaps the most important is the identification of the critical value of the exponent n above which film rupture is not possible. Here, we apply a combination of analytical and numerical methods to further the understanding of this issue, uncovering new types of touchdown behaviour that lead to concrete conjectures regarding the role of n=2 in this type of criticality.

This article is part of the theme issue ‘Science into the next millennium: 25 years on’.

## 1. Introduction

One of the emerging areas of mathematical modelling identified in [1] was the so-called thin-film equation (TFE)


(1.1) $$\begin{array}{lcr} & \frac{\partial h}{\partial t}=\frac{\partial }{\partial x}\left(h^{n}\frac{\partial p}{\partial x}\right),\;p=-\frac{\partial^{2}h}{\partial x^{2}},\;h(x,t)\geq 0,\;n\geq 0, & \end{array}$$

for both its physical and its mathematical implications; see [2], for example, for further background and references. It has indeed been the case that such high-order parabolic equations have amply demonstrated their worth, with wide-ranging investigations pursued—we limit ourselves here to noting that early work includes [3–7]—but a large number of key questions remain open—we seek to shed significant light on one of the most important of these below.

The system of equation (1.1) arises in the surface-tension-driven spreading of a thin film of viscous (Newtonian) fluid over a planar substrate; in this context, the physically most significant values of the exponent n are n=3 (no-slip on the substrate), n=2 (Navier-slip dominated) and n=1 (a slender thread in a Hele-Shaw cell). It is striking that each of these special values also plays an important role mathematically: most famously, n=3 is the main borderline case in terms of the motion of contact points (i.e. points separating regions with h>0 and h≡0); n=2 and n=1 will play important roles in what follows. Other values with n<3 can be viewed as being associated with other slip laws (see appendix A), but the most direct physical interpretations of such cases are perhaps by similarly close analogy with a more general model for power-law fluids [8–11], whereby n>3 and n<3 correspond, respectively, to shear-thickening and shear-thinning flows with no slip (with corresponding equivalences holding either side of n=1). Both of the above (i.e. power-law slip and power-law fluids) lead to a doubly nonlinear generalization to equation (1.1) that is briefly discussed in appendix A.

The most prominent features of equation (1.1) are its degeneracy (i.e. the mobility $h^{n}$ vanishes at h=0 for n>0) and its high order; indeed, it exemplifies many of the difficulties that arise much more generally from these characteristics. The corresponding second-order case (the porous-medium equation, PME), p=h, has been the subject of very extensive analysis (e.g. [12]); a key property of the PME is the presence of a comparison principle, implying in particular that ∂h/∂t>0 at a minimum at which h>0, so a positive solution cannot touch down to h=0. Crucially, this is not the case for equation (1.1) and it is this possibility (having the important physical interpretation of film rupture) that we revisit below (e.g. [13] for an account of early work), an important open question being the upper value of the exponent n for which rupture is possible.

Another area of very extensive current activity is that of non-local diffusion equations, and this provides a further perspective on equation (1.1) as an instinctive model problem. Thus, with a suitably scaled kernel K, the non-local relationship


$$p=\int_{-\infty }^{\infty }\frac{1}{\epsilon }K((x-x^{\prime })/\epsilon )h(x^{\prime },t^{\prime })\;dx^{\prime }=\int_{-\infty }^{\infty }K(X)h(x-\epsilon X,t)\;dX$$

implies, on expanding in powers of ϵ for small ϵ,


$$p∼M_{0}h(x,t)-\epsilon M_{1}\frac{\partial h}{\partial x}(x,t)+\frac{\epsilon^{2}}{2}M_{2}\frac{\partial^{2}h}{\partial x^{2}}(x,t),$$

where $M_{k}\equiv \int_{-\infty }^{\infty }X^{k}K(X)\;dX$ is assumed bounded for k=0,1,2. Then as ϵ→0 the PME results from the first of equation (1.1) unless $M_{0}=0$; if $M_{1}=0$ also (typically because K(X) is even) then a scaled version of equation (1.1) arises when $M_{2}<0$.

A tool widely used in studies of the PME that *is* applicable to the TFE is that of similarity solutions: equation (1.1) has two translation symmetries, $\hat{t}=t+t_{0}$ and $\hat{x}=x+x_{0}$ for arbitrary constants $t_{0}$ and $x_{0}$, and two rescaling symmetries,


(1.2) $$\begin{array}{lcr} & \hat{t}=at,\;\hat{x}=bx,\;\hat{h}=(b^{4}/a{)}^{1/n}h, & \end{array}$$

for arbitrary constants a and b. The symmetries of equation (1.2) imply the existence of a one-parameter (α) family^1^ of similarity reductions


(1.3) $$\begin{array}{lcr} & h=(-t{)}^{\alpha }f(\eta ),\;\eta =x/(-t{)}^{\beta },\;\beta =(1+n\alpha )/4 & \end{array}$$

that will be central in what follows.

The $h\to 0^{+}$ rupture behaviour of equation (1.1) is notoriously complicated and we shall limit ourselves here to the symmetric case in which touchdown occurs at x=0 and t=0 (on appealing to the translation-invariance symmetries) with


$$\text{at}\;x=0\;\frac{\partial h}{\partial x}=\frac{\partial^{3}h}{\partial x^{3}}=0\;\text{for}\;t<0,$$

with equation (1.2) providing a natural candidate for the behaviour as such a singularity develops, though it is far from the only relevant possibility, as we shall see. Aspects of what follows that are of much broader applicability include both (i) the role of symmetries and ‘second-kind’ similarity solutions (e.g. [14]) involving anomalous exponents (α in equation (1.3)), as well as of non-self-similar multiscale descriptions, and (ii) the significance of hierarchies of singular behaviour. We shall return to both of these issues in due course.

## 2. The linear case, n=0

This case is unusually revealing of the range of possibilities despite its linearity; moreover, its tractability provides a valuable starting point for the numerical investigations. That this case is perhaps unexpectedly simple to analyse owes as much to the fact that (exceptionally) h=0 has no special status—so the solution is analytic in both x and t at rupture—as to the TFE being linear when n=0.

Since h=0 at x=0, t=0, with h having a minimum at x=0 immediately beforehand (at least), we have as x→0, $t\to 0^{-}$ that


$$h∼a_{1}x^{2}+a_{2}(x^{4}+24(-t))+a_{3}(x^{6}+360x^{2}(-t))+a_{4}(x^{8}+1680x^{4}(-t)+20160(-t{)}^{2}),$$

where the $a_{i}$ are constants^2^ and successive terms are higher- and higher‐order even polynomials in x, so generic touchdown behaviour has $a_{1},a_{2}>0$ with^3^


(2.1) $$h∼a_{1}x^{2}+24a_{2}(-t)\;\text{for}\;x=O((-t{)}^{1/2}),$$

and there are two singly non-generic cases (unstable to a single mode), namely, $a_{1}=0$, $a_{2}>0$ with


(2.2) $$\begin{array}{lcr} & h∼a_{2}(x^{4}+24(-t))\;\text{for}\;x=O((-t{)}^{1/4}) & \end{array}$$

and $a_{1}>0$, $a_{2}=0$, $a_{4}>0$, with


(2.3) $$\begin{array}{lcr} & h∼a_{1}x^{2}+20160a_{4}(-t{)}^{2}\;\text{for}\;x=O((-t)). & \end{array}$$

Equation (2.2) is the second of a family of similarity solutions of the form equation (1.3), with α=M/2 for integer M=1,2,… and β=1/4, that are candidates for local descriptions of touchdown; the first of these is simply


(2.4) $$h∼a_{1}x^{2},$$

the much more general role of which we shall come to shortly for n≠0. For M odd these similarity solutions vanish at η=0, so an additional term in the expansion (as in equations (2.1), (2.3) and (2.6)) is needed to determine h(0,t) as $t\to 0^{-}$ (conversely for M even there is no $\eta^{2}$ contribution, etc.; such properties are unusual, but intrinsic to the current problem). The cases equation (2.1) and equation (2.3) are *not* of the class equation (1.3) and readily generalize to other n, as we outline below; these are necessarily multiscale—thus equation (2.1) and (2.3) are, respectively, to be supplemented generically by


(2.5) $$\begin{array}{lcr} & h-a_{1}x^{2}∼a_{2}(x^{4}+24(-t))\;\text{and}\;h-a_{1}x^{2}∼a_{3}(x^{6}+360x^{2}(-t))\;\text{for}\;x=O((-t{)}^{1/4}), & \end{array}$$

wherein the right-hand sides are subdominant to each of the terms on the left-hand sides and *are* of the form equation (1.3).

**Figure 1.**
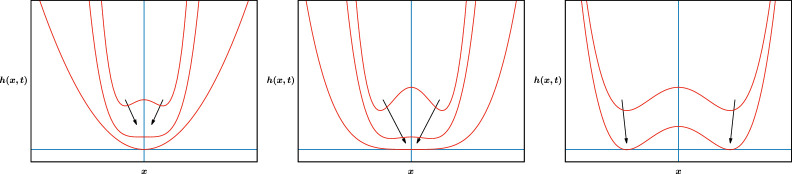
Generic (left and right) and non-generic (centre) touchdown scenarios. (Left) extrema merge before touchdown; (centre) extrema merge at touchdown; (right) separate touchdown points. Arrows indicate the direction of increasing t, successively lower curves corresponding to successively larger values of t.


Interpretations of equations (2.2) and (2.3) are transparent in this context: equation (2.2) lies, for symmetric initial data with (say) a maximum at x=0 and a minimum in each of x>0 and x<0, on the borderline between cases in which the three extrema merge before touchdown (so equation (2.1) ensues) and those in which touchdown occurs simultaneously at points in x>0 and x<0 (see figure 1 for a schematic); equation (2.3) involves touchdown followed immediately by lifting up again, and is thus the borderline between rupture via equation (2.1) and failure to touchdown (with ∂h/∂t reversing sign at x=0, t=0) in the neighbourhood of t=0: simple expressions such as

$$h(0,t)∼(\epsilon -t{)}^{2}-|\epsilon |\epsilon$$

serve to illustrate such behaviour, with ϵ=0, ϵ>0 and ϵ<0, respectively, corresponding to this trichotomy. Such insights have more general implications—indeed, such transitions are not partial differential equation (PDE) specific, being a rather general feature of systems for which touchdown and lift off are possible (cf. [15]).

For completeness, we note that the second (multiscale) hierarchy has


$$h∼a_{1}x^{2}+A_{2M}(-t{)}^{M},\;M=1,2,\ldots$$

for $a_{1},A_{2M}>0$ and that there are other possibilities as exemplified by $a_{1},a_{2}=0$*,* $a_{3},a_{4}>0$ whereby


(2.6) $$\begin{array}{lcr} & h∼360a_{3}x^{2}(-t)+20160a_{4}(-t{)}^{2}\;\text{for}\;x=O((-t{)}^{1/2}); & \end{array}$$

indeed, the general such form involves two positive integers M and L with


$$h∼A_{2M+1}x^{2}(-t{)}^{M}+A_{2L}(-t{)}^{L}\;\text{for}\;x=O((-t{)}^{(L-M)/2})$$

for $A_{2M+1},A_{2L}>0$*,* L>M.

There is an alternative root to the same conclusions when equation (1.1) is posed as an initial value problem on the entire real line, which provides more details and hence is worth recording even though the methodology does not carry over to the nonlinear cases and may not be applicable to other initial boundary value problems for n=0. Introducing the Fourier transform


$$\bar{h}(k,t)=\int_{-\infty }^{\infty }h(x,t)e^{-ikx}\;dx,\;h(x,t)=\frac{1}{2\pi }\int_{-\infty }^{\infty }{\bar{h}}_{0}(k)e^{ikx}\;dk$$

and imposing even initial data


$$h(x,-T)=h_{0}(x),\;\bar{h}(k,-T)={\bar{h}}_{0}(k)$$

for some T>0 such that touchdown occurs at x=0, t=0 we have


$$h=\frac{1}{2\pi }\int_{-\infty }^{\infty }{\bar{h}}_{0}(k)e^{ikx-k^{4}(t+T)}\;dk$$

so that


$$h=\sum_{m=1}^{\infty }c_{m}(-t{)}^{m}\left\{\sum_{n=0}^{m}\frac{1}{(4n)!(m-n)!}\eta^{4n}\right\}-\sum_{m=0}^{\infty }d_{m}(-t{)}^{m+1/2}\left\{\sum_{n=0}^{m}\frac{1}{(4n+2)!(m-n)!}\eta^{4n+2}\right\},$$

where $\eta =x/(-t{)}^{1/4}$ and


(2.7) $$c_{m}=\frac{1}{2\pi }\int_{-\infty }^{\infty }k^{4m}{\bar{h}}_{0}(k)e^{-k^{4}T}\;dk,\;d_{m}=\frac{1}{2\pi }\int_{-\infty }^{\infty }k^{4m+2}{\bar{h}}_{0}(k)e^{-k^{4}T}\;dk$$

determine the coefficients of each of the polynomial similarity solutions in terms of the initial data. Since touchdown occurs at t=0, $c_{0}=0$ holds in equation (2.7).

## 3. n>0: multiscale phenomena

The analysis of this section generalizes^4^ that of the previous one by using the exact solution


(3.1) $$\begin{array}{lcr} & h=x^{2} & \end{array}$$

to equation (1.1) (where we have scaled so that $a_{1}=1$) as a starting point. Aspects of what follows have previously been established (e.g. [13]; it would not be appropriate here to review the extensive literature in the area) but our viewpoint differs, specifically in the way in which we regard equation (3.1) as a similarity solution of the form equation (1.3) with α=1/(2−n), β=1/2(2−n) and viewing the ‘outer’ scale (in the sense of matched-asymptotic expansions) $x=O((-t{)}^{\beta })$ as dictating the singular behaviour.

To set up this outer problem, we must first analyse the ‘inner’ one, in which the right-hand side of the TFE dominates, so that as $t\to 0^{-}$


(3.2) $$h∼h_{0}=x_{0}^{2}(t)+x^{2}\;\text{for}\;x=O(x_{0}),$$

where $x_{0}\ll (-t{)}^{\beta }$ as $t\to 0^{-}$ remains to be determined by matching, the final term in equation (3.2) having been matched into equation (3.1), which is the leading-order outer solution. Setting $h∼h_{0}+h_{1}$, the first correction term is then given by


(3.3) $$\begin{array}{lcr} & \frac{\partial^{3}h_{1}}{\partial x^{3}}=-\frac{2x_{0}{\dot{x}}_{0}x}{(x_{0}^{2}+x^{2}{)}^{n}},\;\frac{\partial^{2}h_{1}}{\partial x^{2}}=\frac{x_{0}{\dot{x}}_{0}}{(n-1)(x_{0}^{2}+x^{2}{)}^{n-1}}-p_{1}(t) & \end{array}$$

for some function of integration $p_{1}(t)$. Hence, for n<3/2


(3.4) $$\begin{array}{lcr} & h_{1}∼-\frac{1}{2}p_{1}x^{2}+\frac{x_{0}{\dot{x}}_{0}x^{4-2n}}{2(n-1)(2-n)(3-2n)}\;\text{as}\;x\to +\infty & \end{array}$$

(we note that n=1, n=3/2 and n=2 are all special cases with respect to equation (3.3), the significance of which will become clearer in due course), while for n>3/2


(3.5) $$\begin{array}{lcr} & h_{1}∼-\frac{1}{2}p_{1}x^{2}+\frac{\pi^{1/2}\Gamma (n-3/2)x_{0}^{4-2n}{\dot{x}}_{0}}{2(n-1)!}x\;\text{as}\;x\to +\infty . & \end{array}$$

Turning now to the outer scale, we set


$$h∼x^{2}+H(x,t)$$

and linearize to give


(3.6) $$\frac{\partial H}{\partial t}=-\frac{\partial }{\partial x}\left(x^{2n}\frac{\partial^{3}H}{\partial x^{3}}\right),$$

with matching into equations (3.2), (3.4), (3.5) determining boundary conditions on equation (3.6), namely,


(3.7) $$\begin{array}{lcr} & \frac{\partial H}{\partial x}=x^{2n}\frac{\partial^{3}H}{\partial x^{3}}=0\;\text{at}\;x=0\;\text{for}\;n<3/2, & \end{array}$$

(3.8) $$\begin{array}{lcr} & H=x^{2n}\frac{\partial^{3}H}{\partial x^{3}}=0\;\text{at}\;x=0\;\text{for}\;n>3/2, & \end{array}$$

the distinction being associated with the difference between equation (3.4) and equation (3.5). The associated eigenmodes take the form


(3.9) $$\begin{array}{lcr} & H=(-t{)}^{\alpha }F(\eta ),\;\eta =x/(-t{)}^{1/2(2-n)} & \end{array}$$

and are to be determined as the non-trivial solutions to


(3.10) $$\alpha F-\frac{1}{2(2-n)}\eta \frac{dF}{d\eta }=\frac{d}{d\eta }\left(\eta^{2n}\frac{d^{3}F}{d\eta^{3}}\right)$$

subject to


$$\frac{dF}{d\eta }=\eta^{2n}\frac{d^{3}F}{d\eta^{3}}=0\;\text{at}\;\eta =0\;\text{for}\;n<3/2,$$

$$F=\eta^{2n}\frac{d^{3}F}{d\eta^{3}}=0\;\text{at}\;\eta =0\;\text{for}\;n>3/2,$$

and to


(3.11) $$F(\eta )=O(\eta^{2(2-n)\alpha })\;\text{as}\;\eta \to +\infty ,$$

in both cases, the possible values of α being the associated eigenvalues.

Determining the other possible contributions as η→+∞ reveals two exponentially growing expressions that are inadmissible (i.e. equation (3.11) amounts to two boundary conditions) and one exponentially decaying one: applying the Liouville–Green method to equation (3.10) requires that Φ(η) in the ansatz


ln⁡F(η)∼Φ(η)asη→+∞

satisfy


$$-\frac{1}{2(2-n)}\eta \frac{d\Phi }{d\eta }=\eta^{2n}{\left(\frac{d\Phi }{d\eta }\right)}^{4}$$

at leading order, so that


(3.12) $$\begin{array}{lcr} & \Phi =-\frac{3}{(2(2-n){)}^{3/2}}\eta^{2(2-n)/3} & \end{array}$$

or


(3.13) $$\begin{array}{lcr} & \Phi =e^{\pm \pi i/3}\frac{3}{(2(2-n){)}^{3/2}}\eta^{2(2-n)/3}, & \end{array}$$

wherein only the real root equation (3.12) is admissible, with both of equation (3.13) being associated with oscillatory growth.

We now address the possible solutions to the linear problem equation (3.6) with equation (3.7) or equation (3.8) as x→0, $t\to 0^{-}$. For n<3/2, the possibilities are given by immediate generalizations of the results for n=0. The first two eigenmodes^5^


(3.14) $$\begin{array}{lcr} & \alpha =0,\;H=a_{0};\;\alpha =1/(2-n),\;H=a_{1}x^{2} & \end{array}$$

should be rejected: the former would dominate equation (3.1) for small x, being associated with t-translation invariance (i.e. with a different touchdown time), while the latter has already been absorbed into the choice of scaling in equation (3.1), reflecting the scaling invariance of equation (1.1). Turning to the remaining eigenmodes, which alongside equation (3.14) are expected to form a complete set in the traditional sense,^6^ the next two are given for n<3/2 (so that equation (3.7) holds) by


(3.15) $$\begin{array}{c} \alpha =1,\;H=a_{2}(x^{4-2n}+4(2-n)(3-2n)(1-n)(-t));\\ \alpha =(3-n)/(2-n),\;H=a_{3}(x^{6-2n}+12(3-n)(5-2n)(2-n)x^{2}(-t)), \end{array}$$

from which the general pattern should be clear; these modes are remarkable both in that those with α=M, $M\in {\mathbb{Z}}^{+}$, have no $x^{2}$ terms, while those with α=M+1/(2−n) omit $x^{0}$ terms, and in that the admissible exponentially decaying term (see equation (3.12)) as η→+∞ is entirely absent. These solutions should be viewed as second-kind similarity reductions of the form equation (3.9) of the linearized problem: that this family of solutions can be constructed explicitly, rather than only numerically, is associated with the sequence of eigenmodes generating a Taylor expansion in (−t) —see appendix A for additional comment and for a generalization for which such a construction is not possible.

The first of equation (3.15) with $a_{2}>0$ gives the generic form of touchdown, with $x_{0}^{2}∼4(2-n)(3-2n)(1-n)a_{2}(-t)$ following on matching. We must defer one of the two singly non-generic cases—with interpretations mirroring those for n=0—to §4; that which generalizes equation (2.3) has $a_{2}=0$, $a_{4}>0$, with $x_{0}\propto \left(-t\right)$ therefore resulting; $a_{3}$ plays no leading-order role in this scenario, but does lead to


$$p_{1}∼-24(3-n)(5-2n)(2-n)a_{3}(-t),$$

which is generically the case.

The above computations closely parallel those for n=0, but an additional check is needed, that is, that H is subdominant to equation (3.1) as $t\to 0^{-}$ on the relevant scale, $x=O((-t{)}^{1/2(2-n)})$, requiring that α>1/(2−n) (i.e. α in equation (3.9) needs to be larger than the value associated with equation (3.1)). Thus, importantly, the first of equation (3.15) is admissible only for n<1, becoming of the same scale as equation (3.1) at n=1, which suggests the possibility that a similarity solution equation (1.3) distinct from equation (3.1) may then come into play, a possibility that we explore below. That the inner ($x=O((-t{)}^{1/2})$) and outer ($x=O((-t{)}^{1/2(2-n)})$) scales would coincide for n=1 provides further evidence for such a possibility. Given the constraint that n<3/2 the other modes are all admissible.

Turning now to the n>3/2 regime, given equation (3.8) the modes with α=M+1/(2−n), $M\in {\mathbb{Z}}^{+}$, starting with the second of equation (3.15) remain applicable, but those with α=M do not, being replaced by ones having α=M+1/2(2−n) of which the first two are


(3.16) $$\begin{array}{lcr} & \begin{array}{c} \alpha =1/2(2-n),\;H=a_{0}x;\\ \alpha =(5-2n)/2(2-n),\;H=a_{2}(x^{5-2n}+4(5-2n)(2-n)(3-2n)x(-t)); \end{array} & \end{array}$$

the first of these must be excluded for similar reasons to those noted above. Admissibility of these requires that α>1/(2−n), so those with α=M+1/(2−n) remain legitimate, while for α=M+1/2(2−n) the constraint


(3.17) $$\begin{array}{lcr} & n<2-1/2M & \end{array}$$

must hold, so in particular the second of equation (3.16), which would otherwise represent the generic scenario, is not viable throughout n>3/2, while equation (3.17) reveals that more and more of these modes become illegitimate as n increases, all being lost at n=2. The minimum height h(0,t) for n<3/2 is determined by matching into the final term of equation (3.5), whereby as $t\to 0^{-}$


$$x_{0}^{4-2n}{\dot{x}}_{0}\propto a_{2M}(-t{)}^{M},$$

so that


$$h(0,t)\propto (-t{)}^{2(M+1)/(5-2n)}.$$

The two most significant outcomes from the above analysis are that (i) touchdown is possible for any n<2 (we recall that n=2 is of specific physical interest), but does so only under more and more exceptional circumstances as n increases, and (ii) there are possible bifurcations (to other solutions of the form equation (1.3)) at n=1 and n=2−1/2M for $M\in {\mathbb{Z}}^{+}$. We turn to the latter next.

## 4. n>0: similarity solutions

Two starting points for the analysis of similarity solutions f(η) of the form equation (1.3) that follows have been identified, namely, (i) by continuation from α=M/2 at n=0^7^ and (ii) via bifurcations at n=1 and n=2−1/2M, where α=1/(2−n). The latter are, in principle, amenable to weakly nonlinear analyses, but these seem unusually involved and we resort from the start to numerical approaches to the boundary value problem:


(4.1) $$\begin{array}{lcr} & \alpha f-\beta \eta \frac{df}{d\eta }=\frac{d}{d\eta }\left(f^{n}\frac{d^{3}f}{d\eta^{3}}\right), & \end{array}$$

(4.2) $$\begin{array}{lcr} & \text{at}\;\eta =0\;\frac{df}{d\eta }=0,\;\frac{d^{3}f}{d\eta^{3}}=0, & \end{array}$$

(4.3) $$\begin{array}{lcr} & \text{as}\;\eta \to +\infty \;f=O(\eta^{\alpha /\beta }). & \end{array}$$

Similarly to equation (3.11), here equation (4.3) amounts to two boundary conditions, with two exponentially growing correction terms excluded. We note that α is an (unknown) eigenvalue (i.e. equations (4.1)–(4.3) represent a nonlinear eigenvalue problem, cf. footnote 1). Our numerical approach (see §5) will adopt a shooting-problem set up from η=0 with


(4.4) $$\begin{array}{lcr} & \text{at}\;\eta =0\;f=1,\;\frac{d^{2}f}{d\eta^{2}}=\gamma , & \end{array}$$

the former exploiting the scaling invariance of equation (4.1), with α and γ both being shooting parameters.^8^

We next outline how we identify solutions to equations (4.1)–(4.3) numerically through the above shooting problem, the solution to which will generically reach zero at a finite value of η, $\eta =\eta_{0}$ say, via^9^


(4.5) $$f∼k_{1}(\eta_{0}-\eta )+k_{2}(\eta_{0}-\eta {)}^{2}+k_{3}(\eta_{0}-\eta {)}^{3-n}\;\text{as}\;\eta \to \eta_{0}^{-},$$

when n<2, wherein the presence of four arbitrary constants $\eta_{0}$, $k_{1}>0$, $k_{2}$ and $k_{3}$ confirms that this behaviour is generic. For special values of α and γ, the solution will reach zero in a singly exceptional (three-parameter) way, with


(4.6) $$\begin{array}{lcr} & f∼k_{2}(\eta_{0}-\eta {)}^{2}+k_{3}(\eta_{0}-\eta {)}^{3-2n}\;\text{as}\;\eta \to \eta_{0}^{-}, & \end{array}$$

for n<1/2, with $k_{2}>0$, and


(4.7) $$\begin{array}{lcr} & f∼K(\eta_{0}-\eta {)}^{3/(n+1)}\text{as}\;\eta \to \eta_{0}^{-} & \end{array}$$

for 1/2<n<3/2, with K>0. The solutions that satisfy equations (4.5)–(4.7) for finite $\eta_{0}$ are inconsistent with equation (4.3),^10^ but those of the form equation (4.6) or equation (4.7) provide the cliffs in the simulations presented below, and hence will be significant in the identification of the desired connections (i.e. solutions that instead satisfy equation (4.3)): these connections are doubly exceptional, having two degrees of freedom (namely, the coefficients of $\eta^{\alpha /\beta }$ and of the exponentially decaying term) as η→+∞, consistent with there being two shooting parameters.

If we suppose $\alpha =\alpha_{*}$, $\gamma =\gamma_{*}$, $f=f_{*}(\eta )$ furnishes a solution to equations (4.1)–(4.3) then for nearby values of the shooting parameters


$$\alpha =\alpha_{*}+\epsilon \alpha_{1},\;\gamma =\gamma_{*}+\epsilon \gamma_{1},\;0<\epsilon \ll 1$$

we can set


$$f∼f_{*}(\eta )+\epsilon F(\eta )\;\text{for}\;\eta =O(1),$$

and demonstrate, for $f_{*}(\eta )∼A\eta^{\alpha /\beta }$ as η→+∞, that


$$F(\eta )∼\eta^{((n-1)\alpha -2\beta )/2\beta }e^{\sigma }\left(\alpha_{1}\kappa_{\alpha }\cos\left(3^{1/2}\sigma +\phi_{\alpha }\right)+\gamma_{1}\kappa_{\gamma }\cos\left(3^{1/2}\sigma +\phi_{\gamma }\right)\right)\;\text{as}\;\eta \to +\infty ,$$

wherein


$$\sigma =3\beta^{4/3}\eta^{1/3\beta }/2A^{n/3},$$

and the constants $\kappa_{\alpha }$, $\kappa_{\beta }$, $\phi_{\alpha }$ and $\phi_{\gamma }$ are independent of $\alpha_{1}$ and $\gamma_{1}$ and could be obtained numerically. Given its exponential growth, ϵF becomes of O(1) under the scaling


(4.8) $$\eta =q(\epsilon )+\delta \xi ,\;q(\epsilon )∼{\left(2A^{n/3}\ln(1/\epsilon )/3\beta^{4/3}\right)}^{3\beta }\;\text{as}\;\epsilon \to 0,$$

with $\delta =(A^{n}q^{(3\beta -1)/\beta }/\beta {)}^{1/3}$, so setting


$$f=Aq^{\alpha /\beta }g,\;\Phi_{\alpha ,\gamma }=(\phi_{\alpha ,\gamma }+3^{1/2}\ln(1/\epsilon ))\mathrm{mod}(2\pi )$$

we obtain, at leading order in 1/ln⁡(1/ϵ), the initial value problem^11^


(4.9)−(g−1)=gnd3gdζ3,(4.10)asζ→−∞,g∼1+eζ/2(α1καcos⁡((3/2)ζ+Φα)+γ1κγcos⁡((3/2)ζ+Φγ)).

The argument by which we identify the desired connections now proceeds in two distinct steps and is of an unusual type. First, for almost all $\left(\alpha_{1},\gamma_{1}\right)$ the solution to equation (4.9) and equation (4.10) will hit zero in the form implied by equation (4.5), but for special curves in the $\left(\alpha_{1},\gamma_{1}\right)$ plane equation (4.6) or (4.7) will apply instead. If we locate such an exceptional zero at $\zeta =\zeta_{*}$, say, then on one side of such a curve the solution will hit zero near $\zeta =\zeta_{*}$, while on the other side it will just fail to do so in this neighbourhood (this being the significance of equation (4.6) and (4.7) as a borderline case) and will instead do so at an O(1) distance further in ζ (with g increasing for an O(1) range of positive $\zeta -\zeta_{*}$); thus these curves will appear as vertical cliffs in surface plots in the (α,γ) plane of the location of the zeros of f.

The second step in the argument then proceeds as follows. Introducing


$$\rho^{2}=(\alpha_{1}\kappa_{\alpha }{)}^{2}+2\alpha_{1}\kappa_{\alpha }\gamma_{1}\kappa_{\gamma }\cos(\Phi_{\alpha }-\Phi_{\gamma })+(\gamma_{1}\kappa_{\gamma }{)}^{2},$$

$$\tan\psi =(\alpha_{1}\kappa_{\alpha }\sin\Phi_{\alpha }+\gamma_{1}\kappa_{\gamma }\sin\Phi_{\gamma })/(\alpha_{1}\kappa_{\alpha }\cos\Phi_{\alpha }+\gamma_{1}\kappa_{\gamma }\cos\Phi_{\gamma })$$

expresses equation (4.10) in the form


$$g∼1+\rho e^{\zeta /2}\cos((\sqrt{3}/2)\zeta +\psi ),$$

and, setting ζ=−2ln⁡ρ+ξ, this becomes


$$\text{as}\;\xi \to -\infty \;g∼1+e^{\xi /2}\cos((\sqrt{3}/2)\xi -\sqrt{3}\ln\;\rho +\psi ),$$

so that g(ξ) depends upon $\alpha_{1}$ and $\gamma_{1}$ only through the combination $\psi -\sqrt{3}\ln\;\rho$ (reflecting the translation invariance of equation (4.9)). Hence, if $\psi =\psi_{c}$, $\rho =\rho_{c}$ lies on a cliff of the type referred to above, then for equation (4.9) and (4.10) the entire logarithmic spiral


(4.11) $$\begin{array}{lcr} & \rho =\rho_{c}e^{(\psi -\psi_{c})/\sqrt{3}} & \end{array}$$

will form the cliff over some range of ψ. In consequence $\alpha_{1},\gamma_{1}\to 0$ as ψ→−∞, so a desired connection necessarily lies at the centre of such a spiral when ψ→−∞ lies in the relevant range.

Two related remarks about this analysis are in order.

(I) The result equation (4.11) is exact for equation (4.9) and (4.10), but only valid asymptotically close to a connection for the original problem equations (4.1), (4.2) and (4.4).
(II) Both the logarithmic dependence of equation (4.8) upon ϵ and the spiral form equation (4.11) militate against the numerics fully uncovering the relevant details but, as we shall see, the above properties suffice for the current purpose.

## 5. Computations of the self-similar solutions

**Figure 2.**
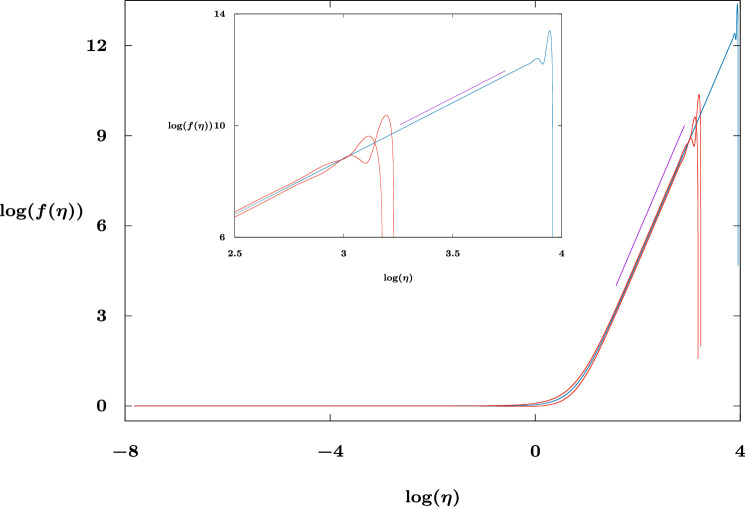
Log–log plots of f(η) versus η for n=0, α=1 with γ=±0.1 (red curves) and γ=0 (blue curve). The line segment has slope 4 (=α/β). The inset plot is an enlargement of the right-hand side of the larger plot.

In this section, we provide numerical support for some of the conjectures made above, moreover determining numerically estimates of the values of $\alpha_{*}$ and $\gamma_{*}$. We numerically solve equation (4.1) and (4.2) with equation (4.4) as a two-parameter shooting problem (α and γ in equation (4.4)) to identify the doubly exceptional solutions that satisfy equation (4.3) as η→+∞.^12^ We present example solutions that demonstrate the effectiveness of the approach in figure 2 for n=0, with α=1 and γ=−0.1,0,0.1. The solutions with γ=±0.1 sit slightly on either side of the exact solution (with γ=0) $f(\eta )=1+\eta^{4}/24$; we include a line segment on the figure to indicate the far-field behaviour $f=O(\eta^{4})$ as η→+∞. We note that the (red) curves in figure 2 (corresponding to γ=±0.1) have f becoming zero well before the curve corresponding to γ=0. Furthermore, we note that the red curves separate from the blue curve in an opposite and oscillatory fashion, this being a consequence of the two exponentially growing oscillatory modes discussed above.

Importantly, numerical errors for the γ=0 solution eventually lead to the growing modes again dominating and as a consequence it can be difficult, even for the linear case, n=0, to distinguish the doubly exceptional solutions. However, as indicated in figure 2, we expect the numerically obtained critical solutions to be the ones that locally (in (α,γ) parameter space) touch down (i.e. have f=0) at the largest value of η and this provides a robust measure for finding such solutions for n>0 also. Our results are therefore primarily presented as (α,γ) parameter surveys of the value of η corresponding to the first zero of f; the sought after exceptional solutions appear (within numerical resolution) at the centre of logarithmic spirals (see §4).

**Figure 3.**
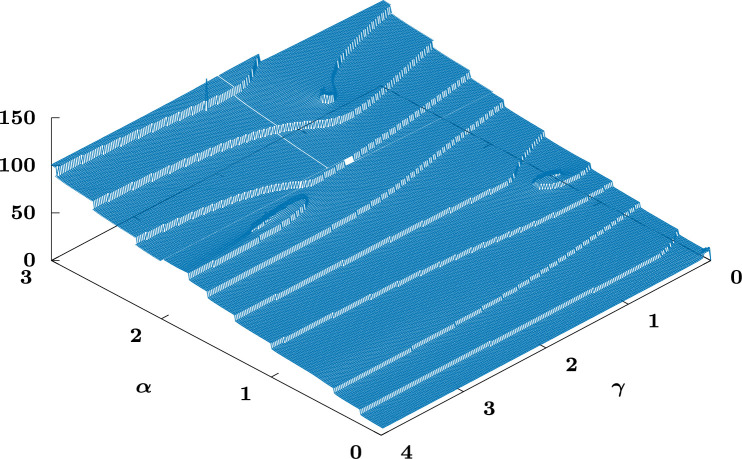
Surface plot for n=0.2, the surface height corresponding to $\eta_{0}$ for $f(\eta_{0})=0$, illustrating the spirals corresponding to the solution branches starting from α=1 (right), 3/2 (left) and 2 (top) at n=0.

**Figure 4.**
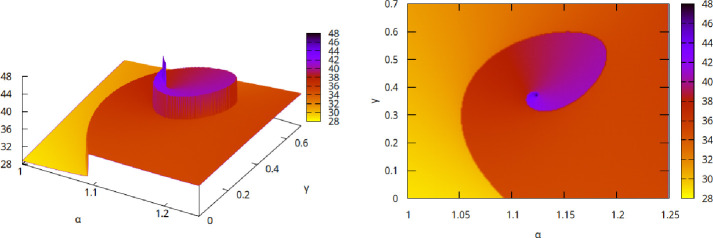
Close-up view of the spiral corresponding to the solution branch starting from α=1 at n=0 from figure 3 (for n=0.2). (Left) side-view, (right) map-view. The darkening colours indicate how $\eta_{0}$ attains a maximum as the centre of the spiral is reached.

Figure 3 shows an example (α,γ) parameter survey for n=0.2. Notably, three doubly exceptional solutions (spiral centres) can be observed, these being the continuations into n>0 of the (rescaled^13^) exact n=0 solutions (i) α=1, $f(\eta )=\eta^{4}+24$, (ii) α=3/2, $f(\eta )=\eta^{6}+360\eta^{2}$ and (iii) α=2, $f(\eta )=\eta^{8}+1680\eta^{4}+20160$ (we omit the figure for n=0). We also include a close-up view of the spiral structure of the branch (i) for n=0.2 in figure 4, wherein it can be observed that $\eta_{0}$ attains a maximum as the centre of the spiral is reached.

**Figure 5.**
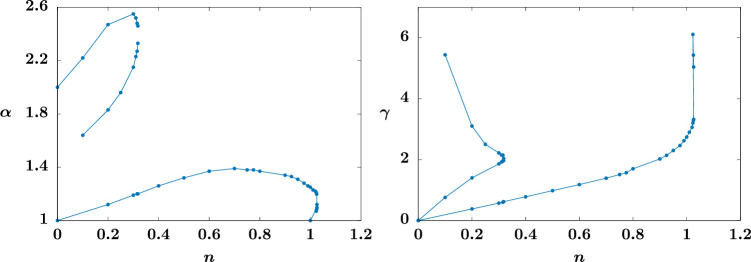
Plots of α (left) and γ (right) against n showing the location of the centre of the spirals located in (α,γ) parameter space (corresponding to doubly exceptional solutions). The highest branch in the right-hand pane corresponds to the middle one on the left, having γ→∞ as $n\to 0^{+}$ (the α=3/2 solution at n=0 having no $\eta^{2}$ contribution).

**Figure 6.**
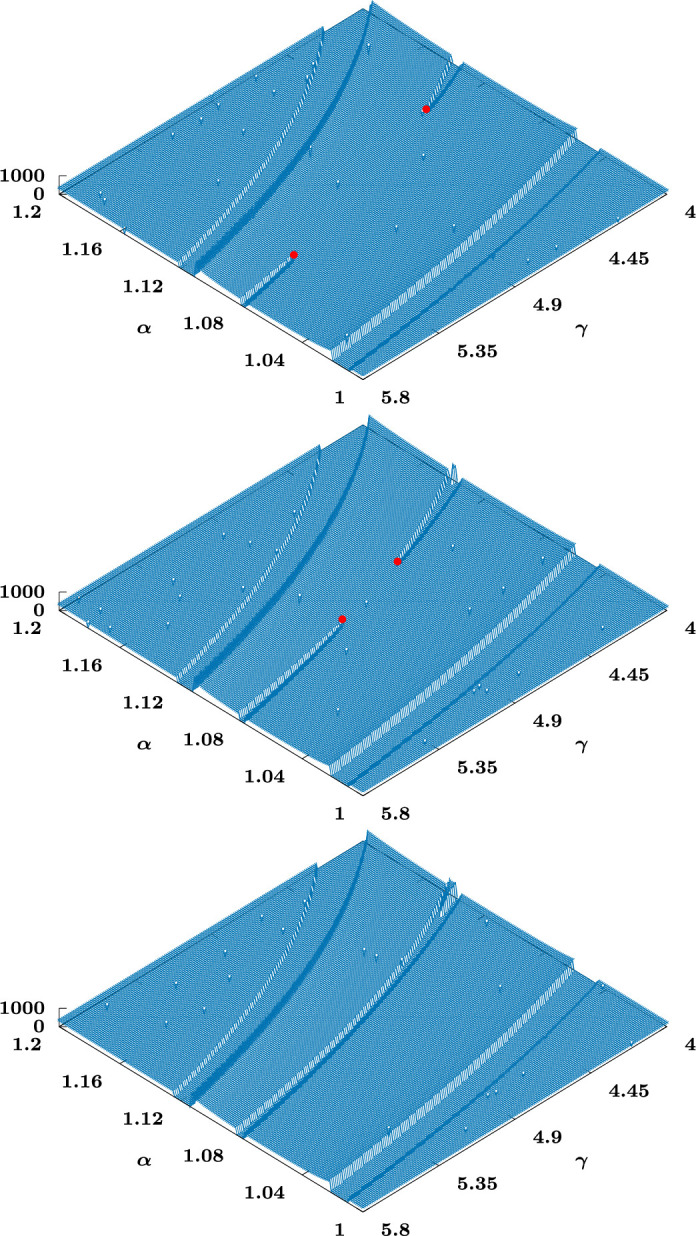
Surface plot for (upper figure) n=1.025, (centre figure) n=1.026 and (lower figure) n=1.027, showing the solution that originates from α=1 at n=0 (upper tip) and the solution that has α=1 at n=1 (lower tip). The surface heights correspond to $\eta_{0}$ for $f(\eta_{0})=0$. The dots are intended to be indicative of the cliff-end tips as they approach each other, with coalescence having occurred by n=1.027.

We record the locations in parameter space of the exceptional solutions as n varies in figure 5. In the left-hand figure, the top branch corresponds to that starting from α=2 when n=0. At n≈0.31, this branch annihilates with that coming from α=3/2 at n=0. In the right-hand figure, the lower branch (corresponding to the one starting from α=1 at n=0) folds back just beyond n=1 before having $n\to 1^{+}$ as γ→∞. In other words, for a value of n close to, but above, one, a fold arises as solutions on separate branches come together, at n≈1.027; given the small range of n beyond n=1 on which the fold occurs, the numerical simulations here are particularly delicate. We include figure 6 to illustrate how the two branches approach each and coalesce as the fold occurs.

The main conclusions from the very extensive numerical study summarized above, which we believe has identified for the first time the role in touchdown behaviour of similarity solutions satisfying equations (4.1)–(4.3), are the following.

(a) The first three similarity solutions identified for n=0 can all be continued into n>0, though the second and third annihilate at a fold at n≈0.32. The first, though, seems to persist until just above n=1, providing the continuation of the singly degenerate solution equation (2.2) the significance of which was noted in §2. The continuation of higher modes would require further numerical study.
(b) The fact that the first branch extends up to n≈1.03 is the single most significant result: the numerical evidence is that a fold occurs there with the solution branch that emerges from n=1, α=1; for α below the value at the fold this branch is expected to be generic in the current context, so extends the class of stable touchdown solutions to the symmetric problem to slightly above n=1. While the above value of n is sufficiently close to one that we cannot exclude this conclusion being the result of numerical inaccuracies, the presence in figure 6 of two terminating cliffs (with termination points that move closer together as the putative fold is approached) provides good qualitative evidence for their being two distinct, but neighbouring, solutions in the relevant range of n. In any case, the implications of the singly unstable solution branch noted in the context of equation (2.2) makes its presence up to at least n=1 noteworthy.
(c) No solutions to equations (4.1)–(4.3) that bifurcate off α=1/(2−n) at n=2−1/2M have been detected, the implication being that the bifurcations are plausibly to similarity solutions describing other types of singular behaviour (associated with contact-point motion). Such matters warrant further investigation.

## 6. Discussion

We start with some comments specific to the above analysis, before turning to some more general matters. The most significant result above is that touchdown scenarios are available up to, but we believe not including, n=2, but in the symmetric case rupture seems to be possible generically only to slightly above n=1, both classes of analyses undertaken above being needed to establish this claim. That non-generic scenarios are available for larger n is mathematically significant, but such phenomena would naturally be hard to capture numerically from time-dependent solutions and would compound the challenges associated with rigorous studies. Our results are based on a combination of formal and numerical approaches and are accordingly not in that sense conclusive (including because we have not addressed asymmetric cases, in which other balances are known to be relevant); nevertheless, the evidence they provide for new touchdown scenarios up to, but not beyond, n=2 would seem to be significant given both the long-standing open nature of the question and the physical significance of n=2 (there is additional evidence that 2<α/β<4/n should hold in the case of equation (1.3), reinforcing this claim of criticality). With regard to n=2, we should note that the combination of nonlinearities


(6.1) $$\begin{array}{lcr} & \frac{\partial h}{\partial t}=-\frac{\partial }{\partial x}\left((h^{3}+\lambda h^{2})\frac{\partial^{3}h}{\partial x^{3}}\right) & \end{array}$$

is commonly adopted in applications, with the dimensionless slip parameter λ often small— nevertheless, in the limit h→0 with which we are concerned, the $h^{2}$ term dominates the right-hand side even for small λ.

Criticality is indeed a common theme in the above, with the presence of a number of distinct regimes in the exponent n being of particular significance. Analysis of the critical cases (notably n=1, n=3/2 and n=2) is, as usual, particularly delicate and has not been undertaken here. While the analyses above establish local spatial profiles at touchdown, we have also not outlined the resulting post-touchdown evolution, though much of this can be gleaned from [16].

The following more general issues seem worth highlighting. Self-similarity and other symmetry arguments are central to progress in such applications; moreover, the results above are in fact of near-direct broader relevance to other singular phenomena in the TFE. Entire hierarchies of the type described above necessarily arise in such contexts, for reasons implicit in the discussion of the singly non-generic cases in §2. A combination of various analytical and numerical approaches, as exploited above, is of very broad applicability, the interplay between them being particularly fruitful. A variety of analytical arguments were needed to characterize the possibilities in full, and it is striking that a number of the most instructive ones are elementary, allowing us to elaborate many of their details—a further example of this type, which also revisits the issue of critical exponents, relates to the possible local behaviours of H in equation (3.6), namely, time-dependent multiples of


$$x^{0},\;x^{1},\;x^{2},\;x^{3-2n},$$

the comparison of the last of these with the other three immediately identifying n=3/2, n=1 and n=1/2 as critical.

As is typical of such studies, the formal and numerical arguments could provide the basis for subsequent rigorous approaches, involving analysis either of the (high-dimensional) phase space of the ODE (4.1) or of the full (time-dependent) PDE (1.1). Each would be valuable and each challenging; their combination would be particularly potent.

A number of natural extensions suggest themselves. The critical exponents could change in higher dimensions (the two-dimensional generalization being the only physically meaningful one in the thin-film context), though a preliminary analysis of the radially symmetric case suggests that surprisingly little changes (including the upper bound of n=2); non-radially symmetric cases are, unsurprisingly, much more involved, though aspects seem to be accessible to methods closely related to those above (most notably, the steady state solution equation (3.1) generalizes in two dimensions to $ax^{2}+by^{2}$ for arbitrary positive constants a and b, allowing non-radial singularities to be identified). The inclusion of additional physics, such as con/disjoining pressure terms and stochastic effects (e.g. [17]) seems likely to have more dramatic implications for criticality and goes beyond the scope of the current study.

In summary, we have identified new touchdown scenarios relating to a long-standing open problem, with the critical value n=2 of the exponent in equation (1.1) deserving emphasis. We have applied a combination of analytical and numerical arguments, with intuition as an invaluable adjunct, in deriving two classes of second-kind self-similar behaviour: first, similarity solutions to the linearized problem equations (3.6), (3.9), are the key ingredients in §3; second, and most notable, those of the full PDE (1.1), satisfying equations (4.1)–(4.3), have been explored in §§4 and 5–to the best of our knowledge the role of such solutions in touchdown has not previously been identified, though the regimes in which they *are* present are perhaps surprisingly limited. The story is certainly not yet over, with intriguing issues remaining—open questions have been noted above, and other approaches would be needed to rule in or out dynamics more exotic than the power-law dependencies uncovered above—but it seems worth highlighting that considerable insight can be obtained by perhaps deceptively simple-looking arguments.

## Data Availability

The data used in creating the figures included in this article are available in the supplementary material [18].

## Footnotes

^1^
Here α is an arbitrary real number. Once reductions of the form equation (1.3) are deployed in the analysis of specific initial value problems its admissible values become constrained, often to a countably infinite set (as in §4, for example).

^2^
The $a_{i}$ are independent arbitrary constants (subject to sign constraints noted below). For the Cauchy problem they can be determined in terms of the initial data using Fourier transforms, being proportional to the relevant $c_{m}$ and $d_{m}$ in equation (2.7).

^3^
This demonstrates without more ado the lack of a comparison principle: h is decreasing at a minimum.

^4^
Aspects of the results are also applicable to n<0, but we shall focus exclusively on n≥0.

^5^
We express each of these in terms of H since derivation from the PDE (3.6) seems more transparent than from the ordinary differential equation (ODE), ODE (3.10), reflecting the invariance of equations (3.6)–(3.8) under t translations, for reasons outlined in appendix A: they can readily be rewritten in terms of F(η) as in equation (3.9).

^6^
Some insight into the reasoning behind this claim is provided in appendix A.

^7^
Because f(0)=0 for M odd, it is not clear whether those with M odd can be continued with f(η)>0; a related issue arises in continuation from n=2−1/2M.

^8^
The shooting problem from η=+∞ is more delicate to set up but has been used numerically to home in on relevant regimes of α and γ.

^9^
A minor modification is needed for n=1.

^10^
For $k_{3}=0$, equation (4.6) could be continued past $\eta =\eta_{0}$, but this would involve the presence of a zero in h prior to t=0, contrary to the prescription of t=0 as the touchdown time; correction terms to equation (1.3) could in principle maintain h>0 up to t=0, but such a scenario would involve an overspecified problem for f(η) and would in any case be picked up in the course of the numerics.

^11^
Correction terms to q, beyond the expression in equation (4.8), are needed to scale out the arbitrary constant prefactor that would otherwise multiply the ζ-dependent terms in equation (4.10).

^12^
The shooting computations employ a fixed-step fourth-order Runge–Kutta solver written in FORTRAN. The code was validated against the analytical results herein and against other implementations of the Runge–Kutta methodology.

^13^
The α=3/2 solution has f=0 at η=0, so the shooting problem is initiated at a small positive n and the branch continued from there.

## Appendix A. Doubly nonlinear generalization

Here we consider the generalization

(A 1)
$$\begin{array}{lcr} & \frac{\partial h}{\partial t}=-\frac{\partial }{\partial x}\left(h^{n}{\left|\frac{\partial^{3}h}{\partial x^{3}}\right|}^{m-1}\frac{\partial^{3}h}{\partial x^{3}}\right),\;m>0, & \end{array}$$

of equation (1.1) introduced in [8], first to substantiate a remark in §1 and, second, both to illustrate that the above results generalize rather readily and to indicate how, in this broader context, equation (1.1) is exceptional in certain respects. We stress that the analysis that follows is not intended to be complete.

Equation (A 1) was derived in [8] as the generalization of equation (1.1) to power-law fluids (shear-thinning for m>1 and shear-thickening for m<1) in the cases n=m+2 (Stokes flow) and n=1 (Hele-Shaw flow). A further application arises in the slip-dominated case for a power-law generalization of the Navier-slip law, whereby (in the notation of [8])

(A 2)
$$\begin{array}{lcr} & \lambda \mu \frac{\partial u}{\partial z}=-|u{|}^{s-1}u,\;s>0 & \end{array}$$

holds on the substrate z=0, λ being the slip coefficient, with equation (A 2) equating the shear stress to a power of the tangential velocity. Following the usual lubrication-theory procedures then in the slip-dominated case leads after rescaling to equation (A 1) with n=(s+1)/s, m=1/s, so n=m+1; often in such applications the right-hand side of the PDE would be taken to include both no-slip (see Stokes flow above) and slip terms (cf. equation (6.1) above).

A significant critical exponent for m=1 above was n=2, which can be understood as being the exponent at which $h∼P(t)x^{2}$ ceases to be a legitimate local balance as x→0. This immediately generalizes to n=(m+3)/2 as follows: the correction term in the extended local expansion

$$h∼Px^{2}-\frac{m^{3}(3P^{n}{)}^{-1/m}|\dot{P}{|}^{(m-1)/m}\dot{P}}{\left(3m+3-2n)(2m+3-2n)(m+3-2n\right)}x^{(3m+3-2n)/m}$$

requires

(A 3)
$$\begin{array}{lcr} & n<(m+3)/2 & \end{array}$$

for self-consistency.

The inner balance corresponding to the first of equation (3.3) is, for ${\dot{x}}_{0}<0$,

$$\frac{\partial^{3}h_{1}}{\partial x^{3}}=\frac{(-2x_{0}{\dot{x}}_{0}x{)}^{1/m}}{(x_{0}^{2}+x^{2}{)}^{n/m}},$$

so that possible contributions to $h_{1}$ as x→∞ are proportional to

$$x^{0},x^{1},x^{2},x^{(3m+1-2n)/m},$$

a generalization to n=3/2 as a critical exponent for m=1 then follows on comparison of the second and fourth of these to be given by

(A 4)
$$\begin{array}{lcr} & n=(2m+1)/2 & \end{array}$$

with the outer problem (cf. equation (3.6))

(A 5)
$$\begin{array}{lcr} & \frac{\partial H}{\partial t}=-\frac{\partial }{\partial x}\left(x^{2n}{\left|\frac{\partial^{3}H}{\partial x^{3}}\right|}^{m-1}\frac{\partial^{3}H}{\partial x^{3}}\right) & \end{array}$$

accordingly being subject to

(A 6)
$$\begin{array}{lcr} & \text{at}\;x=0\;\frac{\partial H}{\partial x}=x^{2n}{\left|\frac{\partial^{3}H}{\partial x^{3}}\right|}^{m-1}\frac{\partial^{3}H}{\partial x^{3}}=0\;\text{for}\;n<\min((2m+1)/2,(m+3)/2)), & \end{array}$$

(A 7)
$$\begin{array}{lcr} & \text{at}\;x=0\;H=x^{2n}{\left|\frac{\partial^{3}H}{\partial x^{3}}\right|}^{m-1}\frac{\partial^{3}H}{\partial x^{3}}=0\;\text{for}\;(2m+1)/2<n<(m+3)/2, & \end{array}$$

with the latter range being present only for m<2.

The first eigenmode for equation (A 6) takes the form (cf. equation (3.15))

(A 8)
$$\begin{array}{lcr} & H=a_{2}\left(x^{(3m+1-2n)/m}+{\left(\frac{\left(3m+1-2n)(2m+1-2n)(m+1-2n\right)}{m^{3}}\right)}^{m}(-t)\right) & \end{array}$$

for n<(m+1)/2: a sign change would be needed for n>(m+1)/2 but in any case self-consistency here requires (3m+1−2n)/m>2, so that

(A 9)
$$\begin{array}{lcr} & n=(m+1)/2 & \end{array}$$

generalizes the n=1 critical exponent above and equation (A 8) is inapplicable for larger n. The generalization of the second of equation (3.15) is

(A 10)
$$\begin{array}{lcr} & H=a_{3}\left(x^{(3m+1-2n)/m}+3{\left(\frac{\left(3m+3-2n)(2m+3-2n)(m+3-2n\right)}{m^{3}}\right)}^{m}x^{2}(-t)\right), & \end{array}$$

which is relevant to both equation (A 6)) and (A 7) and is self-consistent whenever equation (A 3) holds. Finally, in generalizing equation (3.16) in the case of equation (A 7) we have

(A 11)
$$\begin{array}{lcr} & H=a_{2}\left(x^{(3m+3-2n)/m}+2{\left(\frac{\left(3m+2-2n)(2m+2-2n)(m+2-2n\right)}{m^{3}}\right)}^{m}x(-t)\right), & \end{array}$$

requiring

(A 12)
$$\begin{array}{lcr} & n<(m+2)/2 & \end{array}$$

for the exponent in the first term on the right-hand side to be greater than two.

This analysis thus highlights several features specific to the case m=1 with which we have been largely concerned, these having broader ramifications. First, because equation (A 5) is nonlinear the remaining eigenmodes cannot be explicitly constructed (it is indeed noteworthy that equations (A 8), (A 10) and (A 11) can be). For m=1 the eigenmodes can be viewed as representing a Taylor expansion in (−t): this can be interpreted as a consequence of the facts that H=0 plays no distinct role in equation (3.6) due to the invariance of equation (3.6) under translations in H, and that ∂H/∂t is a solution whenever H is, satisfying the boundary conditions as well as the PDE; more generally, however, integrating or differentiating in t does *not* map solutions of equation (A 5) into one another and, while H=0 still has no special status, ∂H/∂t=0 does not because $\partial^{3}H/\partial x^{3}$ can then in general be expected to be singular in (−t), consistent with the higher modes no longer being explicit. While the modes for equation (3.6) (exceptionally) decouple (in the sense that those with $H\propto x^{0}$ or $x^{1}$ as x→0 form hierarchies separate from those with $H\propto x^{2}$) this cannot be expected to be the case more generally. Second, for m=1 the critical exponents in equations (A 4) and (A 12) coincide, which simplified the discussion somewhat. The surprising implication is that for (m+1)/2<n<(2m+1)/2, equation (A 8) is unacceptable, implying that the anticipated forms of touchdown associated with equation (A 5) are non-generic, but from equation (A 11) such touchdown is reinstated as generic for (2m+1)/2<n<(m+2)/2.

In summary, the following regimes are suggested by the above considerations. For n>(m+3)/2 touchdown is not possible, encompassing the entire range of no-slip Stokes flow (n=m+2) and s<1, m>1 for the slip law equation (A 2). For n<min((2m+1)/2,(m+3)/2)*,* equation (A 6) applies but the *generic* touchdown mode equation (A 8) is available only for n<(m+1)/2, while equation (A 11) similarly potentially implies *generic* touchdown for m<1, (2m+2)/2<n<(m+2)/2. Thus for Hele-Shaw such touchdown is postulated to be possible for, curiously, m<1/2 and m>1, while generic touchdown of this type is prohibited for nonlinear slip, though non-generic forms are not excluded for m<1.

This discussion does not touch on full similarity solutions generalizing those considered in §4, which we leave as a fully open problem, alongside investigation of the higher eigenmodes^14^ for equation (A 5) (subject to equation (A 6) or equation (A 7) alongside the exclusion of exponential growth as x→+∞) for m≠1.
